# Targeting Angiogenesis in Pancreatic Neuroendocrine Tumors: Resistance Mechanisms

**DOI:** 10.3390/ijms20194949

**Published:** 2019-10-08

**Authors:** Javier Pozas, María San Román, Teresa Alonso-Gordoa, Miguel Pozas, Laura Caracuel, Alfredo Carrato, Javier Molina-Cerrillo

**Affiliations:** 1 Medical Oncology Department, University Hospital Ramon y Cajal, 28034 Madrid, Spain; pozas.jav@gmail.com (J.P.); marasanroman@gmail.com (M.S.R.); pozas.javier@gmail.com (M.P.); laura.caracuel@salud.madrid.org (L.C.); acarrato@telefonica.net (A.C.); 2 The Ramón y Cajal Health Research Institute (IRYCIS), CIBERONC, 28034 Madrid, Spain; 3 Alcalá University, 28805 Madrid, Spain

**Keywords:** neuroendocrine tumors, antiangiogenic, resistance, sunitinib

## Abstract

Despite being infrequent tumors, the incidence and prevalence of pancreatic neuroendocrine tumors (P-NETs) has been rising over the past few decades. In recent years, rigorous phase III clinical trials have been conducted, allowing the approval of several drugs that have become the standard of care in these patients. Although various treatments are used in clinical practice, including somatostatin analogues (SSAs), biological therapies like sunitinib or everolimus, peptide receptor radionuclide therapy (PRRT) or even chemotherapy, a consensus regarding the optimal sequence of treatment has not yet been reached. Notwithstanding, sunitinib is largely used in these patients after the promising results shown in SUN111 phase III clinical trial. However, both prompt progression as well as tumor recurrence after initial response have been reported, suggesting the existence of primary and acquired resistances to this antiangiogenic drug. In this review, we aim to summarize the most relevant mechanisms of angiogenesis resistance that are key contributors of tumor progression and dissemination. Furthermore, several targeted molecules acting selectively against these pathways have shown promising results in preclinical models, and preliminary results from ongoing clinical trials are awaited.

## 1. Introduction

Neuroendocrine neoplasms (NEN) are a diverse group of tumors mainly arising from the diffuse endocrine cells derived from the neural crest. These tumors have a complex clinical and biological behavior, which varies depending on the place of origin, the hormone production, and the histological differentiation. Among them, P-NETs make up 12% of all gastroenteropatic neuroendocrine tumors (GEP-NETs) and 3% of primary pancreatic tumors [1]. The incidence of P-NETs has increased from 0.18 cases per 100,000 inhabitants in 1986 to 0.48 cases per 100,000 inhabitants in 2012. In terms of survival, patients with metastatic P-NETs disease present a median overall survival (OS) of about 3.6 years [2] and 5-year survival rate of 30.2%. Nowadays, 65% of P-NETs are diagnosed as unresectable or metastatic disease, with a consequent worse prognosis [3,4,5].

Among the different available therapeutic options and considering molecular biology in pNET, antiangiogenics agents, such as sunitinib, play a central role. The aim of this review is to make a comprehensive overview about molecular pathways involved in P-NETs, focusing on angiogenesis and its mechanisms of resistance, from the basis to the clinic.

## 2. Neuroendocrine Tumors Classification

Histological tumor classification is a cornerstone in NEN treatment. This classification has been recently modified with the last update in 2017 (Table 1). The main modifications were the ki67 cut off levels, which changed from 2 to 3%, and the concept of MANEC (mixed adeno-neuroendocrine carcinoma) that developed into MENEN/MINEN (Mixed endocrine non-endocrine neoplasms). The other novelty was the inclusion of high grade well-differentiated (WD) neuroendocrine tumor (NET G3). Previously, all tumors with a high proliferation index (Ki67 > 20%) were considered neuroendocrine carcinomas (NEC) regardless of the level of differentiation [6].

NETs can also be classified in two major categories: functioning and non-functioning. Functioning NETs produce a wide spectrum of clinical syndromes depending on which hormone is released. Non-functioning tumors, which correspond to 50–85%, may secrete some substances but they do not cause a hormonal syndrome [7].

On one side, functioning tumors cause different symptoms depending on the amine or peptide hormone released; insulinomas cause hypoglycaemias, which result in unusual behaviour, confusion, trembling, and diaphoresis. Gastrinomas are related to Zollinger-Ellison syndrome, causing peptic ulcer disease and diarrhea. Glucagonoma is associated to necrolytic migratory erythema, diabetes, and diarrhea. The clinical syndrome of VIPoma is watery diarrhea with hypokalaemia and hypochlorhydria [4].

On the other side, non-functioning (NF) tumors can be subdivided into three types: NF-P-NETs that do not produce hormones; NF-P-NETs that secrete hormones at a low enough level not to cause symptoms; and NF-P-NETs that produce hormones such as chromogranin, neuron-specific enolase, pancreatic polypeptide or ghrelin, which do not produce a clinical syndrome. The presence of these three subtypes may lead to a delayed diagnosis [8].

## 3. Treatment

For unresectable or metastatic NETs, the treatment is multidisciplinary and includes systemic and loco-regional therapies. SSAs are nowadays the front-line therapy in NETs (Table 2): They are effective in palliating hormone-related symptoms and have demonstrated, in phase III trials, their role as antiproliferative agents, delaying disease progression in patients with NETs. Current practice guidelines are based on the results of the PROMID and CLARINET trials. The PROMID study randomized 85 patients with WD midgut NETs to receive placebo or octreotide LAR 30 mg intramuscularly in monthly intervals until progression or death. The study closed early due to slow accrual, but achieved the benefit in terms of time to progression (14.3 months in the octreotide arm vs. 6.0 months in the placebo arm; HR0.34, *p* < 0.001) [9]. This study was the first trial to demonstrate the antitumoral effect of SSA in patients with WD midgut G1 NET (Ki67 1–2%). In this sense and to assess the benefit of SSA in a wider population, the CLARINET trial evaluated the role of lanreotide depot 120 mg every 28 days compared with placebo in patients with advanced WD GEP-NET and a Ki67 < 10%. The study was positive for the experimental arm, with a primary endpoint of median PFS not reached (65.1% vs. 33% in the placebo group at 2 years) [10].

Even though SSAs remain the first line treatment of most patients with unresectable WD NETs, the majority of patients will eventually experience disease progression. Ongoing research about the use of peptide receptor radionuclide therapy (PRRT) with radiolabelled SSAs, such as octreotide or octreotate in P-NETs, are showing promising results in somatostatin receptor (SSTR)-expressing tumors. This therapy is composed by a carrier molecule (SSA), a radionuclide isotope (^111^In, ^90^Y and ^177^Lu), and a chelator [Tetra-azacyclododecane-tetra-acetic acid (DOTA) and diethylenetriamine penta-acetic acid (DTPA)] that binds and stabilizes the complex. So far, the most satisfactory results have been obtained with ^177^Lu-based radiolabelled SSAs, based on the phase III trial NETTER 1 [11]. However, research is going further, looking for radionuclides that offer higher affinity, greater efficacy, and less toxicity [12].

Chemotherapy also has a role in the treatment of patients with advanced WD NETs. The use of streptozocin (SZT) alone or in combination with doxorubicin or fluoropirimidines has been the standard chemotherapy approach for many years [5]. Temozolomide has also shown activity among P-NETs in combination with other drugs, such as thalidomide and capecitabine. In a study where all subtypes of NETs were included, the efficacy of the combination of capecitabine and temozolomide (CAPTEM) in metastatic P-NETs was demonstrated [13]. There is an ongoing phase II clinical trial using the above-mentioned combination, with a median PFS in the P-NETs cohort of 18.2 months [14].

The role of chemotherapy in the treatment sequence of patients with P-NETs is currently under research in the SEQTOR trial (NCT02246127) that randomizes patients to receive everolimus followed by STZ-fluorouracil (5-FU) vs. STZ-5FU followed by everolimus. In addition, the preliminary results of an ongoing phase II trial comparing CAPTEM versus temozolomide alone in advanced P-NETs, reported a prolonged PFS (22.7 months) [15]. However, these results may be influenced by an imbalance between the two arms, since P-NETs in the CAPTEM subgroup had a lower grade [16].

Targeted agents have also broken in the therapeutic landscape of patients with P-NETs. Considering the expression of vascular endothelial growth factor receptors (VEGFR), platelet-derived growth factor receptor (PDGFR) α and β, and stem cell factor receptor (c-kit) in P-NETs, antiangiogenic agents such as sunitinib, pazopanib, cabozantinib, or lenvatinib are or have been under research in this group of patients showing considerable antitumoral activity. Sunitinib is the only one approved for P-NETs treatment at the moment (Table 3) [5]. The mTOR pathway is proven to be involved in tumor development and progression of P-NETs by over-activating mTOR. Because of this reason, everolimus, a rapamycin analogue mTOR inhibitor, has been investigated in this group of patients based on preclinical data. Indeed, the phase III randomized clinical trial RADIANT 3 showed that everolimus had an impact in median PFS (11.4 vs. 5.4 months; HR 0.35, *p* < 0.0001) when compared to placebo in the treatment of advanced P-NETs [17].

Waiting for definitive biomarkers to guide therapeutics decisions, the treatment algorithm in P-NETs depends on histological classification, radiological images, and clinical symptoms, as well as patient comorbidities. In this sense, treatment selection at each moment of the patient disease is crucial in order to obtain the maximal benefit with every agent administered. Future and current translational research may help in deciding which treatment strategy may be the most effective in a more accurate manner. [24].

Nevertheless, initial approaches are aimed to define molecular pathways involved in tumor development and resistant mechanisms to previous therapies that may guide therapeutic decisions. As we have previously mentioned, angiogenesis is a key path in P-NETs progression, and antiangiogenic agents, such as sunitinib, have obtained a significant survival benefit. In this sense, the improvement in the biological knowledge of resistance mechanisms to antiangiogenics would be relevant for novel therapies development that may improve patients’ survival.

## 4. Molecular Biology

In recent years, there has been an international effort to improve the understanding of the molecular biology of P-NETs. It is really important to properly classify tumors according to their aggressiveness in order to individualize the treatment approach. Most P-NETs are sporadic, but some of them occur as part of some hereditary endocrinopathies: Multiple Endocrine Neoplasia type I (MEN1), Von Hippel Lindau syndrome, Neurofibromatosis type 1 (NF1), and Tuberous Sclerosis Complex (TSC). Patients with MEN1 will develop a P-NET in 80 to 100% of the cases, whilst the other syndromes have a lower incidence (<20%). However, somatic mutations of MEN1 occur in over a third of P-NETS [25].

In a cohort involving 59% of early stage and 41% of metastatic P-NETs, mutations were identified in genes involved in chromatin remodelling such as MEN1, DAXX and ATRX as well as those implicated in mTOR pathway (PTEN and TSC2). It was noticed that tumors that have mutations in both MEN1 and either ATRX or DAXX have a 100% OS at 10 years, whilst in the absence of this mutation, 60% die within 5 years [24]. This data was confirmed in a retrospective evaluation performed by Yachida S. et al. that also noted that RB1 and TP53 alterations are largely restricted to PD NECs, with no changes identified in WD NETs [26].

More recently, whole-genome sequencing was performed in 102 primary P-NETs (18.4% of them were metastatic). Four molecular pathways were found to be involved in the development of these tumors [27]:

(1) DNA damage repair: Germline mutations seem to have an important role in the development of P-NETs; several germline-damaging variants of the base-excision-repair *MUTYH* gene and the homologous recombination genes *BRCA2* and *CHEK2* have been reported.

(2) Chromatin remodelling: Inactivating mutations of *MEN1*, *SETD2*, *ARID1A*, and *MLL3* lead to widespread transcriptional dysregulation.

(3) Telomere maintenance: Approximately one third of P-NETs express inactivating mutations in *DAXX* or *ATRX*, which correlates with telomere length and repeat content, hence resulting in a poorer prognosis,

(4) mTOR signalling activation: Several inactivating mutations have been described in inhibitors of the mTOR signalling pathway, such as *PTEN*, *TSC1*, *TSC2*, *EWSR1,* and *DEPDC5*. These mutations are related with poor prognosis in some patients and could be used as new prognostic response factors in the future.

## 5. Angiogenesis in P-NETs and Drug Development: Sunitinib

Angiogenesis is a biological process regulated by pro-angiogenic and anti-angiogenic factors and, among these, VEGF and PDGF are particularly relevant. VEGF binds to tyrosine-kinase receptors (TKRs) including Flt1 (VEGFR1), KDR/Flk-1 (VEGFR2), and Flt-4 (VEGFR3). These TKRs are expressed on the membranes of endothelial cells, vascular smooth muscle cells, and monocytes/macrophages. The production of VEGF is regulated by local oxygen availability. The hypoxia-inducible factor-1 (HIF-1) binds to VEGF, promoting and stimulating gene transcription and mRNA stability. HIF1 has two subunits: HIF1α and HIF1β. Under normoxic conditions, HIF1α is hydroxylated on proline residues by prolyl-hydroxylase domain (PHD) proteins and then binds to von Hippel-Lindau protein (VHL), allowing ubiquitination by an E3 ubiquitin ligase complex and further proteasome degradation. Notwithstanding, in hypoxic conditions, proline residues are not hydroxylated. Therefore, they are not recognized by VHL, hence translocating to the nucleus and dimerizing with HIF1β, finally binding to ‘hypoxia responsive elements‘(HRE) which are specific DNA sequences located in promoter regions of target genes. This process results in the transcription of several genes involved in proliferation, angiogenesis, survival, and apoptosis. These genes include: VEGF, PDGFβ, transforming growth factor α (TGF-α), erythropoietin (EPO), matrix metalloproteinase 1 (MMP-1), epidermal growth factor receptor (EGFR), hepatocyte growth factor receptor (HGFR/cMET), cyclin D1, stromal cell-derived factor 1 (SDF1), and its receptor CXC chemokine receptor 4 (CXCR4). It also promotes the transcription of genes that induce anaerobic metabolism, such as GLUT-1, hexokinase, and PDK-1 5. The other important regulatory element is PDGF, which has an 18–24% homology with VEGF and binds to PDGFα and β. These two TKRs are expressed mainly in pericytes [28,29].

Particularly in NETs, there is a high demand of vascularization for nutrient delivery to the growing tumor. WD-NETs are highly vascularized by significant upregulation of HIF1α. The activation of HIF1α is driven by genetic inactivation of VHL protein and the stimulating hypoxic conditions that are typically present in P-NET cellular environment. Chromogranin A (CgA) is a protein that is commonly expressed and secreted by P-NETs and its positivity on IHQ is a diagnostic hallmark of GEP-NETs, while serum CgA is used for follow up. This circulating biomarker has been associated with angiogenesis. Also, WD P-NETs being highly vascularized tumors, they express high levels of VEGF, VEGFR-2 and 3, and PDGFR α and β. When the tumor dedifferentiates, VEGF expression is lost and vascularization density decreases, which is a paradox found in P-NETs. Nonetheless, no association between VEGF expression and OS has been proved [30].

**Figure 1:**
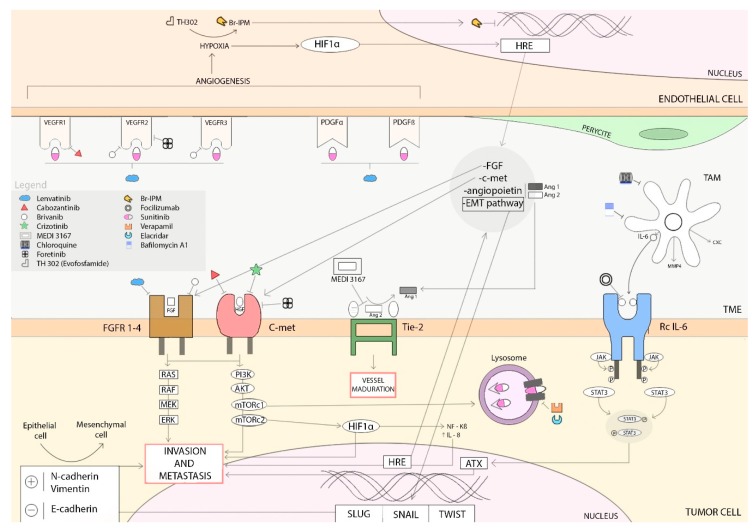
Overview of antiangiogenic drug targets and mechanisms of resistance described. In this figure, we illustrate the most relevant mechanisms of resistance to sunitinib: hypoxia-induced activation of alternative proangiogenic pathways (FGFs, c-MET, EMT activation or angiopoetins), autotaxin upregulation, and sunitinib-induced autophagy. Different agents targeting these mechanisms of resistance are represented, some used in daily practice and some under clinical development (Arrows define activation).

Sunitinib malate is a molecule designed to compete with ATP for binding within the intracellular domain of tyrosine kinase vascular endothelial growth factor receptors 1–3 (VEGFR 1–3); platelet-derived growth factor receptors α and β, (PDGFRs-α/β); stem-cell growth factor receptor (CD117/KIT); fms-related tyrosine kinase 3 (CD135/FLT3); colony-stimulating factor 1 receptor (CD115/CSF1R), rearranged during transfection receptor (RET); and the glial cell line-derived neutrophic factor receptor (GDNF) [31] (Figure 1).

Through this mechanism of action, sunitinib has proven to reduce endothelial cell density and pericyte coverage of tumor vessels, hence delaying tumor growth [32].

Sunitinib has demonstrated activity in advanced, progressive, and WD P-NETs. The efficacy of this drug was established initially in phase I and II trials [18,33]. Later on, the phase III clinical trial SUN1111 comparing sunitinib with placebo, showed an improvement in PFS (11.4 vs. 5.5 months) and OS (median not reached, but the observed HR favoured sunitinib), being then approved by the regulatory agencies for the treatment of P-NETs [19].

Despite the proven efficacy of sunitinib, there have been numerous reports of early progression as well as tumor recurrence after an initial response, suggesting the existence of both primary and acquired resistances, which may compromise the use of this therapy [34] and poses a clinical challenge, providing the rationale for further research in the underlying mechanisms of resistance in order to circumvent this clinical problem. Preclinical GEP-NET models have been used to elucidate these resistance mechanisms to current therapies as well as to cope with them and evaluate new therapies [35]. Different in-vivo models are available, the most used being the promoter of rat insulin gene-2 (RIP), which drives the transgenic expression of simian virus 40 (SV40) large T antigen (Tag).

It is important to underline that resistance to sunitinib seems to be transient and reversible in several cases, with a possible use of sunitinib as a rechallenge in the clinic after a period of “off-sunitinib treatment”. Based on this fact, there is a recruiting phase II clinical trial (RESUNET, NCT02713763) which aims to evaluate the efficacy of a rechallenge with sunitinib in advanced or metastatic WD P-NETs which have already progressed to sunitinib in previous lines. 

## 6. Resistance Mechanisms to Antiangiogenic Treatments

### 6.1. Hypoxia-Induced Activation of Alternative Proangiogenic Pathways and Metastasic Dissemination

#### 6.1.1. HIF-1α Pathway

**Figure 2:**
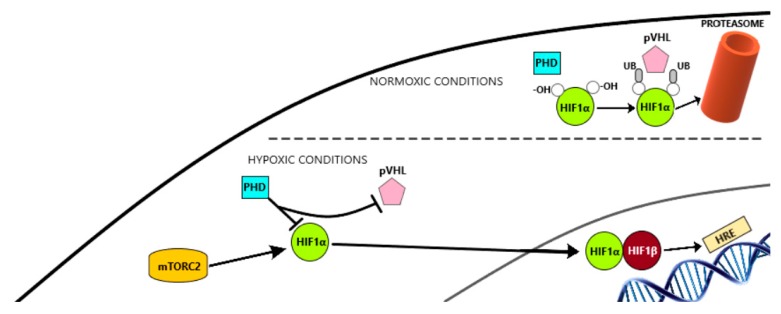
HiF 1α modifications depending on oxygen concentration in cells.

An established cause for the development of resistance to VEGF-targeted therapy is tumor hypoxia. HIF-1α is a transcription factor induced by hypoxic conditions and is the key driver of angiogenesis in P-NETs (Figure 2). 

There are two mechanisms that can lead to HIF-1α activation. Firstly, the VHL gene can be inactivated by genetic or epigenetic alterations, either because of germline mutations as part of VHL disease or because of somatic mutations in sporadic P-NETs. Secondly, HIF-1α activation can result from tumor hypoxia. Over time, hypoxia appears in the central areas of bulky tumors, followed by spontaneous necrosis. Alternatively, TME hypoxia can also be the consequence of treatment with antiangiogenic agents such as sunitinib. Therefore, HIF-1α activation represents at the same time sensitivity to therapeutic targets, by activation of VEGF/VEGFR and mTOR pathways, and resistance to them, by activation of alternative pathways associated with the expression of proangiogenic factors, such as FGFs, ephrins, angiopoietins, EMT, or c-MET [34].

Several hypoxia-targeted therapies have been under development.TH-302 (evofosfamide) is a hypoxia-activated prodrug which is metabolized to its active form, bromo-isophosphoramide mustard (Br-IPM), under hypoxic conditions. TH-302 acts as an alkylant of DNA, causing the death of the cell. In addition, it can also diffuse, surrounding the hypoxic territory into the normoxic territory and is able to eradicate these tumor cells as well. There are several ongoing studies in different tumors, including a phase II trial (SUNEVO) in combination with sunitinib in patients with previously untreated metastatic WD-P-NETs. Unfortunately, recent results were reported, failing to demonstrate activity in terms of tumor shrinkage, finding 2/17 responders (11.8%) and stable disease achieved in 76.5% of patients [36].

It was demonstrated that, under prolonged treatment with sunitinib, initial tumor growth inhibition was followed by tumor progression with a more invasive behaviour and a high density of microvessels. This vascularization came from the different angiogenesis pathways mentioned before (VEGF-independent cascades) [37]. It should be pointed out that the expression of semaphorin 3A (Sema3A), a well-known component of FGF-induced angiogenesis, has demonstrated in monotherapy, similarly to sunitinib, to inhibit tumor growth. In contrast to sunitinib, Sema3A also prevents tumor invasion and dissemination to distant organs. Moreover, thanks to its vascular normalizing activity, Sema3A ameliorates blood vessel function and improves cancer tissue oxygenation through its ability to induce normoxia, thus counteracting the TME hypoxia created by sunitinib-induced activation of HIF-1α, EMT, and c-MET. Maione et al. proved that the combination of sunitinib and Sema3A synergistically enhanced RIP-Tag2 mouse survival by inducing less invasive and less frequent neoplasms in these models. Then, it has been suggested that the combination of these two agents may represent an interesting therapeutic strategy but further studies are required [38].

FGF levels have been proven to be related to VEGFR inhibitors resistance. Inhibiting FGF signalling with a FGF-trap led to a significant decrease of tumor burden and angiogenesis. Brivanib is a first class dual VEGFR2-3/FGFR1-2-3 tyrosine kinase receptor inhibitor (TKI) which has shown activity after failure of first line VEGFR inhibition in RIP-Tag2 mice in comparison with anti-VEGFR2 monotherapy (DC101) or anti-FGF ligand capture (FGF-Trap). Using brivanib in monotherapy resulted in a more enduring tumor stasis and vascular inhibition, with promising results as the first line but also second line in the context of the failure of VEGF pathway inhibitors (Sunitinib). Furthermore, an early switch to the second line with early signs of revascularization could avoid the manifestation of evasive resistance [39].

Furthermore, lenvatinib is a TKI which binds to VEGFR1-3/FGFR1-4 as well as PDGFRα, KIT, and RET, providing tumor growth control and inhibiting neoangiogenesis and lymphangiogenesis. It also acts as an immune modulator in TME and may cope with resistance to antiangiogenic drugs through FGFR 1-4. In the phase II TALENT clinical trial, lenvatinib was used in two cohorts: P-NETs and gastrointestinal tract NETs (GI-NETs) with ORR as primary endpoint. Up to date, TALENT has proved the highest ever reported ORR by central radiology assessment, being 40.4% in P-NETS, with a promising PFS, but is yet to be published [21].

Angiopoietin/tie2 pathway is suggested to promote angiogenesis through VEGFR inhibition. In clear cell renal cell carcinoma (ccRCC), the expression of angiopoietin-2 (Ang2) was prominent in patients treated with sunitinib, ascending when the tumor was progressing [40]. These findings have also been observed in P-NETs. Angiopoietin 2 is a context-dependent agonist of Tie2. In the absence of VEGF, it inhibits Ang1/Tie2 signalling, which plays a key role in the maturation and stabilization of the vasculature, leading to vascular regression and endothelial cell death. In the presence of VEGF, it leads to remodelling and sprouting of blood vessels, exposing endothelial cells to VEGF. MEDI3617 is a monoclonal antibody targeting Ang2 which has shown effectiveness in inhibiting tumor growth in different tumor xenograft models. Nonetheless, it enhances hypoxia and upregulates the different pathways mentioned above [41]. Combining anti-Ang2 antibody with VEGF-targeted therapies is an interesting research field with results still being awaited.

#### 6.1.2. Epithelial-Mesenchymal Transition (EMT)

EMT is a biological process in which a polarized epithelial cell undergoes several biochemical changes in order to switch into a mesenchymal cell phenotype, including enhanced migratory capacity, invasiveness, elevated resistance to apoptosis, and greatly increased production of extracellular matrix proteins [42]. Therefore, EMT plays an important role in tumor progression and metastasis. As we stated before, hypoxia-induced HIF-1α activation seems to trigger alternative signalling pathways including EMT. There are some EMT-inducing signals such as HGF, EGF, PDGF, and TGF-β emanating from the tumor-associated stroma or tumor microenvironment (TME), that activate several transcription factors in cancer cells, notably Snail, Slug, and Twist, leading to subsequent suppression of specific adhesion molecules such as E-cadherin. Sunitinib-treated tumors have a high expression of Snail and the mesenchymal markers vimentin and N-cadherin; whereas the expression of E-cadherin is inhibited. Therefore, we can infer that VEGF blockade by sunitinib enhances invasiveness by activating EMT [38]. The significance of Snail and E-cadherin expression in P-NETs has been evaluated using human tissue samples in a retrospective study. It was found that patients with low Snail expression and preserved E-cadherin expression had a significantly lower risk of vascular or lymphatic invasion, lymph node involvement or liver metastasis, as well as a lower WHO classification, when compared to the group containing patients with low Snail and reduced E-cadherin, high Snail and preserved E-cadherin expression, and high Snail and reduced E-cadherin [43].

Treating mice RIP-Tag2 tumors with sunitinib highly increased NF-Kβ expression. Since it activates HIF-1α and promotes EMT, cancer invasion, and tumor angiogenesis, it may represent another mechanism of resistance to sunitinib. NF-Kβ also orchestrates the tissue inflammatory response induced by hypoxia, including leukocyte infiltration. Further studies are required to properly address this topic [38].

Recently, Ikezono and co-workers discovered that doublecortin-like kinase 1 (DCLK1), a marker for intestinal and pancreatic cancer stem cells, has a robust and ubiquitous expression in P-NETs. DCLK1 induces p-FAK/SLUG-mediated EMT [44]. Therefore, targeting DCLK1 may be a novel therapeutic approach against P-NETs.

#### 6.1.3. c-Met Activation

C-Met or hepatocyte growth factor receptor (HGFR) is a tyrosine-kinase receptor encoded by proto-oncogene MET. The endogenous ligand of c-Met is HGF. The HGF/c-Met axis is involved in tumor growth, migration, and metastasis and has become an important therapeutic target in various cancers. Hypoxia enhances c-Met expression in tumor cells through HIF-1α binding sites on the c-MET promoter, resulting in an increased tumor growth, proliferation, and invasion [45].

In a preclinical study, after 1 week of treatment with sunitinib, c-Met staining was strong and ubiquitous in tumor cells of Rip-Tag2 mice. Also, c-Met expression was higher when tumor cells were exposed to hypoxia in vitro. In this study, they proved that concurrent treatment with sunitinib and c-Met inhibitor crizotinib reduced tumor invasion and metastasis. Furthermore, the treatment of Rip-Tag2 mice with dual VEGF and c-Met inhibitor cabozantinib showed similar outcomes [46]. Also using the Rip-Tag2 mouse model, multi-TKI foretinib demonstrated comparable results [47].

Sema3A, alone or in combination with sunitinib, inhibits the Met expression and phosphorylation and participates in the reduction of tumor spreading and metastasizing, which is useful to overcome this evasive resistance [38].

In ccRCC, VHL inactivation induces overexpression and activation of c-MET and AXL. Also, there is evidence that not only MET, but also AXL expression levels increase during chronic treatment with sunitinib, which is associated with a poor outcome. Cabozantinib is a VEGFR, c-MET and AXL inhibitor that is under clinical trial in P-NETs (Phase III CABINET Trial), yet results have to be awaited. Cabozantinib effectively supresses the expression and activation of AXL and MET, hence impairing sunitinib treatment-induced prometastatic behaviour in cell culture and overcoming resistance to sunitinib in xenograft models [48].

### 6.2. Alternatives Modes of Vascularization

P-NETs can exhibit alternative pathways of vascularization which are weakly dependent on the VEGF pathway. Vascular co-option is the process by which tumor cells develop around normal vessels, stealing their oxygen and basic nutrients, without the need for angiogenesis. This process has been described in a mouse model of islet cell carcinogenesis [37]. In this report, animals that were treated with anti-VEGFR2 antibodies for 4 weeks showed an increase in vessel density. Also, the presence of co-opted vessels was confirmed. It is known that there is an initial regression of co-opted vessels, probably as a defence mechanism. In the centre of the neoplasm, tumor cells are organized around these few co-opted vessels without any angiogenic response, resulting in a massive cell tumor death. However, in the periphery of the tumor, there is very active angiogenesis. All of this suggests that tumors may originate as an avascular mass that co-opts with pre-existing vessels, and later the surviving peripheral cells activate angiogenesis. The expression of Ang2 seems to be the principal regulator of this process: The absence of VEGF facilitates vessel regression but its presence favours angiogenesis [37]. Hence, targeting both VEGF and Ang2 could be an option to overcome vascular co-option.

When there is no VEGF stimulation, host blood vessels can split into new vessels without the need for endothelial proliferation. This is a very fast process called intussusceptive angiogenesis. First, there is a migration of endothelial cells of opposite walls into the lumen of the vessel, contacting each other and forming a transluminal bridge. Secondly, there is a reorganization of the inter-endothelial cell junctions and a perforation of the bilayer allowing growth factors to penetrate into the lumen. Thirdly, the interstitial pillar is formed and filled with pericytes and myofibroblasts, providing an extracellular matrix. Lastly, the interstitial pillar grows and the endothelial cells retract, allowing the formation of two separate blood vessels [49]. Since there is no cell proliferation involved in this process, anti-proliferative agents are unlikely to overcome this mechanism of resistance.

Vascular mimicry refers to the plasticity of aggressive tumor cells which are able to masquerade endothelial cells and form de novo vascular networks. This process is associated with a malignant phenotype and a poor clinical outcome [50]. The presence of vascular mimicry has been found as part of multiple microvascular alterations in mouse models of pancreatic neuroendocrine tumor [51]. This process is being largely studied and some proteins are potential target therapies, with a current phase II trial using CVM-1118 (NCT03600233) in advanced NETs, including P-NETs, refractory to standard therapy.

### 6.3. Hypoxia-Induced Recruitment of Bone-Marrow Derived Cells

Several bone marrow-derived cells (BMDCs) are recruited due to the local hypoxic tumor environment. Furthermore, hypoxia due to vessel regression after antiangiogenic therapy also increases the recruitment of BMDCs, such as vascular progenitors, pro-angiogenic monocytic cells, monocytes, VEGFR-1-expressing hemangiocytes, and other myeloid cells. These cells locally promote the formation of new microvessels, maintaining the high-demanded blood supply of tumoral tissue. On the other side, VEGF inhibitors induce the release of several cytokines which recruit BMDCs allowing the formation of a premetastatic niche environment. Furthermore, these cytokines induce changes in the epithelium that facilitate adhesion, permeability, and egression of tumor cells from the blood vessels [52].

### 6.4. Inflammatory Cells Infiltration: Tumor-Associated Macrophages

Nowadays, there is robust evidence supporting the key role of TME as a modulator of tumor progression. Healthy stromal cells not only facilitate disease progression but also alter the response to different therapeutic strategies, notably those involving angiogenesis. Among these cells, we find lymphoid and myeloid lineages, including in this last group, tumor-associated macrophages (TAM). TAM can be divided into M1 macrophages and M2 macrophages. The former secrete pro-inflammatory cytokines with anti-tumoral effect while the latter induce anti-inflammatory signals that can facilitate tumor progression, hence worsening prognosis in various tumors, including P-NETs. Specifically, the infiltration of TAMs within primary P-NETs is associated with an increased proliferation, presence of liver dissemination, and disease recurrence after surgery [53].

Anti-VEGF agents’ efficacy can be impaired by pre-existing inflammatory cells within the tumor niche that produce various factors which can promote angiogenesis, thus overcoming the VEGF pathway blockade. Among these inflammatory cells, TAMs modulate the TME by secreting several factors, such as MMP-9, CXC chemokines, and pro-inflammatory cytokines (IL-6, TNF-α, IL-1), as well as VEGF, which are all mediators of angiogenesis. On one hand, the overexpression of the above-mentioned factors leads to angiogenesis via alternative pathways; on the other hand, the upregulation of VEGF may mitigate the blockade effect of targeted inhibitors, inducing the so-called angiogenic switch, leading to enhanced microvessel formation and tumor progression [54].

Krug and co-workers recently investigated the impact on tumor progression and metastasis of TAMs [53]. They found that TAM infiltration in metastasis was considerably higher compared to primary tumor tissues. Similarly, poor differentiation (G3 neuroendocrine carcinomas) was associated to a higher TAM infiltration when compared to well-differentiated tumors (*p* < 0.0001). Using a genetically modified mouse model (RIP-Tag2), the association between TAM infiltration and P-NET progression was assessed. The results showed that TAM infiltration (measured in F4/80+ cells / 10 HPF) increased from 7 (in hyperplasic tumors) to 35 (in angiogenic tumors) and 85 (in invasive tumors).

They also investigated the effect of targeting TAMs with liposomal clodronate in the genetic P-NET mouse model RIP-Tag2. This molecule was proven to significantly impair angiogenesis by depleting TAMs, hence decreasing tumorigenesis. However, the influence of liposomal clodronate on tumor progression and growth did not reach statistical significance, which may suggest that TAMs are particularly relevant in the early stages of tumorigenesis. Moreover, dual targeting of angiogenesis by the simultaneous administration of sunitinib and liposomal clodronate in RIP-Tag2 mice failed to show synergistic antiangiogenic efficacy (although there was a significant reduction of microvessel density and infiltrating TAMs when compared to monotherapy with sunitinib) [53,54]. This lack of synergism may be explained because liposomal clodronate leads to a downregulation of VEGF, and sunitinib also affects predominantly VEGF-dependent angiogenic pathways.

### 6.5. Increase of Pericyte Coverage

Pericytes are smooth muscle-like cells embedded in the basement membrane of small blood vessels and capillaries. There are several markers such as PDGFRβ, α-smooth muscle actin (SMA), regulator of G-protein signaling-5, and desmin, that allow us to identify pericytes, distinguishing them from other cell types. Tumor-associated pericytes are loosely attached to the endothelium and express an atypical expression of markers. These cells are an important cellular regulator in tumor angiogenesis and a pathological activation of them induces the formation of abnormal complicated microvessel networks embedding the tumor cells [55,56]. The PDGF family is composed of four polypeptide chains that assemble into five dimeric isoforms (PDGF-AA, PDGF-BB, PDGF-AB, PDGF-CC, and PDGF-DD) which bind to two tyrosine kinase receptors (PDGFRα and PDGFRβ). These proteins are implicated in pericyte coverage and can also regulate tumor vascularization. Endothelial cells release PDGF-BB that binds to PDGFRβ which is expressed by pericytes [57]. Therefore, PDGFRβ stimulates cell migration and proliferation. In fact, they play a key role in proliferation, survival and motility during tumor growth and invasion [58]. It has been found that using PDGFRβ inhibitors to target tumor-associated pericytes, along with standard antiangiogenic therapies, reduces pericyte coverage of blood vessels, showing a synergic efficacy. This process has been reported in a genetically engineered mouse model of P-NETs [59].

It is known that increased pericyte-generated microvessel formation confers to antiangiogenic treatment resistance in ccRCC but, paradoxically, a lowered pericyte population impairs tumor vascular network but also increases the likelihood for metastatic dissemination. Due to this complicated balance, treatment with antiangiogenic agents should be carried out sensibly, since there is a tight space between inhibiting tumor growth and promoting tumor progression [60].

### 6.6. Autotaxin Upregulation

As we stated before, pre-existing TAMs can express several factors that promote angiogenesis and protect tumor vessels from VEGF blockade. Among these molecules, interleukin 6 (IL-6) plays an important role. IL-6 was found to contribute to the progression of several malignancies by enhancing the expression of angiogenesis-related genes such as VEGF [61,62,63]. IL-6 is a key factor in the STAT3 signalling pathway; IL-6 is recognized by its specific receptor which induces JAK proteins to bind to it. JAK then activates the phosphorylation of gp130 at several tyrosine residues, where STAT3 binds to. Then, STAT3 is phosphorylated, dimerized and translocated into the nucleus, finally allowing the transcription of target genes [64].

Autotaxin (ATX) has been reported to be a target of STAT3 transcriptional regulation in breast cancer [65]. The importance of ATX in P-NETs has been recently evaluated. ATX expression was found to be significantly higher in P-NET tissues with elevated IL-6 levels when compared to normal pancreatic parenchyma. Furthermore, high ATX expression was associated with higher tumor grade, TNM staging and lymph node metastasis. P-NET tumor cells were transfected with ATX siRNA, which downregulated ATX expression, significantly inhibiting the metastatic capacity of P-NET cells. Similarly, transfecting tumor cells with STAT3 siRNA, decreased protein levels of STAT3 and ATX. All of this suggests that upregulation of ATX in P-NET via IL-6-induced STAT3 activation, correlates with the metastatic capacity of P-NET cells [66]. Recently, a pathologic analysis of 144 non-metastatic primary GEP-NETs who underwent curative-intent resection showed that high STAT3 expression is associated with an increased Ki67 index, presence of lymphovascular invasion, and worse 3 years relapse-free survival [67].

In ccRCC, an increase in IL6 levels was found under treatment with sunitinib, which leads to the activation of AKT/mTOR and STAT3 signalling, VEGF expression, and TKI resistance. High IL6 levels were also connected with a shorter PFS and OS. By using a humanized antihuman IL6 receptor tocilizumab, sensitivity to sunitinib was reinstated [68].

### 6.7. Sunitinib-induced Autophagy

Lysosomal sequestration is a process in which hydrophobic weak base compounds travel freely across the lysosomal membrane due to their hydrophobic nature, being protonated inside the lysosome and therefore not being able to exit across the lysosome membrane again [69]. Most TKIs are membrane-permeable weak bases and can be trapped in the acidic lysosomal compartment, not being able to access to their target zone and decreasing drug concentration at the intracellular target site. In the case of sunitinib, there is an ABC transporter P-glycoprotein (P-gp) implicated in this process. The use of P-gp inhibitors, such as verapamil or elacridar, in ccRCC preclinical models, enhances sunitinib antitumor activity [70,71].

Also, since the pH gradient between the acidic luminal pH of the lysosome and the cytoplasm is essential in this process, the loss of efficacy of sunitnib can be reversed with agents that alkalinize lysosomes such as bafilomycin A1, a H^+^-ATPase inhibitor. However, this agent is too toxic for its use in vivo, and choloroquine is an available alternative that has shown promising results in P-NET preclinical studies in combination with sunitinib [72].

### 6.8. Overexpression of EZH2

The overexpression of histone methyltransferase enhancer of zeste homologue 2 (EZH2) promotes tumor angiogenesis by inactivating antiangiogenic factor through methylation and is one of the causes of resistance to sunitinib, being reversible upon dose escalation, suggesting a dynamic adaptation of tumors. Also, using an inhibitor of EZH2 has been shown to increase the activation of tumor suppressor and re-establish sensitivity to sunitinib in both cell lines and PDX models [73,74].

### 6.9. IL8 Serum Levels

In P-NETs patients under treatment with sunitinib, IL8 levels increase almost two-fold over baseline and tend to return to the baseline concentration during the following 2 weeks off treatment. This high concentration is associated with shorter OS and PFS [75].

Under sunitinib treatment, IL8 upregulation is mediated through a HIF1α-independent manner, via NF-kβ, and it leads to activate angiogenesis in endothelial cells through CXCR2 and, consequently, VEGFR2, leading to persistent angiogenesis. Thus, targeting both VEGF and IL8 signalling or even NF-Kβ signalling may be a good mechanism to overcome this type of resistance.

Huang et al. demonstrated an up-regulation of IL8 on the development of resistance to sunitinib in ccRCC cell lines, as well as a correlation between high baseline IL8 concentrations and primary resistance to sunitinib treatment in patients’ samples. Using IL8-neutralizing antibodies, there was a recuperation of sensitivity to sunitinib in the resistant mice. This data may be extrapolated to P-NETs, but further research is required [76].

### 6.10. Placental Growth Factor

Placental growth factor (PlGF) is a VEGF-homolog implicated in tumor angiogenesis, thereby promoting tumor growth, progression, and dissemination. It has been proposed that PlGF stimulates angiogenesis through several mechanisms. Firstly, PlGF binds to Flt1 (VEGFR1) and to neuropilin-1 (NRP1) and -2 (NRP2), but not to Flk1 (VEGFR2). Then, PlGF acts as a “decoy receptor” for VEGF acting as a negative regulator for VEGFR2. This increases the bio-availability of VEGF to stimulate VEGFR-2, stimulating endothelial cell migration, growth and survival, and increasing the proliferation of cancer-associated fibroblasts and smooth-muscle cells. Secondly, it is thought that when PIGF binds to VEGFR 1, angiogenesis is stimulated through the activation of several intracellular signalling pathways. A third mechanism involves the PIGF/VEGFR-1-complex-mediated transphosphorylation of VEGFR-2 which amplifies VEGFR-2 signalling. Also, PIGF induces angiogenesis by up-regulating the expression of VEGF-A, FGF2, PDGFβ, and MMPs and by recruiting BMDCs [77].

Circulating and tumoral PlGF is found to be elevated in patients with P-NETs in comparison with control serum and respective healthy tissue. Its importance relays on the fact that high PlGF levels are associated with reduced tumor-related survival and/or shorter time-to-progression. Furthermore, PlGF is also found to be induced as a result of antiangiogenic therapies and conventional chemotherapy, being a relevant mechanism of resistance to take into account. These data make PlGF a possible potential therapeutic target, a prognostic marker itself, and also a response marker for selected therapy [77].

## 7. Overview

Treatment goals in P-NETs combine the improvement of survival, relief of tumor-related symptoms, inhibition of tumor growth, prevention of tumor complications, and maintenance of a good quality of life. As it has been mentioned before, the optimal sequence of treatment is under debate; nowadays, the decision of the treatment is based on clinical and histological characteristics, according to evidence-based scientific results.

The better biological knowledge of carcinogenesis of P-NETs is expected to lead to the discovery of prognostic or predictive biomarkers that could help to individualise the sequential treatment and development of new agents. Nonetheless, cancer cells may adapt to these new agents and develop different mechanisms of resistance, posing a challenge to overcome them and offering new lines for further research.

Although there are different mechanisms of resistance to sunitinib already identified, tumor hypoxia seems to be the most relevant, since it leads to tumor progression by activating several pathways. To date, no selective HIF-1α inhibitor has been approved as anti-tumor therapy. No other therapies against specific targets that are activated through HIF1α have been approved yet. It is important to underline the role of FGF, with Brivanib as a promising novel therapeutic agent. Furthermore, Sema3A has demonstrated as an important role, inhibiting tumor growth and dissemination and counteracting sunitinib-induced hypoxia. Combination of these agents with sunitinib needs further investigation to establish their benefit.

Several multi-TKIs with combined anti-VEGF and anti-MET activity have shown increased angiogenesis inhibition and suppressed tumor invasion and metastasis in P-NET models. This has led to the development of randomized clinical trials. There is currently a phase II study to evaluate the response rate of cabozantinib in advanced pancreatic neuroendocrine and carcinoid tumors, and a phase III CABINET study, which will evaluate the PFS in 395 patients with advanced or metastatic or not surgically resectable NETs after first line systemic treatment progression. Other potential lines of investigation involve the combination of sunitinib with crizotinib, a c-MET inhibitor, or using multi-TKI such as foretinib.

Alternative ways of vascularization which do not depend on VEGF pathway stimulation are important for better understanding the physiopathology of these tumors. Firstly, aberrant vessel development, which depends on Ang2 among others, may be coped with by using a dual blockage of VEGF and Ang-2. Secondly, vascular intussusception is thought to be increased through VEGFR blockade. However, since there is no cell proliferation involved in this process, it is unlikely that anti-proliferative agents can overcome this mechanism of resistance. The development of new agents targeting the migration of endothelial cells may be the key to effectively control intussusceptive angiogenesis. Finally, vascular mimicry stands out for its lack of response to angiogenesis inhibitors. However, many proteins that facilitate this process have been described, so we can consider some of them as potential therapeutic targets. For instance, the molecular compound CVM-1118 is a potent anti-cancer agent that inhibits vascular mimicry formation, and is currently being tested in a phase II clinical trial, which results have to be awaited.

The importance of TME as a modulator of tumor progression is highly known. Special consideration is given to TAMS, with several ongoing lines of investigation: Clodronate has proven to decrease tumorigenesis in P-NETs, and the development of clinical trials using this molecule in monotherapy or in combination with current standard therapies may lead a new therapeutic approach in P-NETs.

Pericyte coverage plays a delicate role in tumor progression since the increase of these cells, induced by the treatment with sunitinib, may be associated to the development of resistance to it, although this is still controversial. On the other side, the loss of pericyte coverage is associated with an increased metastatic dissemination. Using PDGFRβ inhibitors to target tumor-associated pericytes, along with standard antiangiogenic therapies, may help to reach an adequate balance.

As we have already mentioned, pre-existing TAMs are related to an autotaxin upregulation through IL-6 mediation, activating the STAT3 pathway. Published data seems to indicate that this upregulation of ATX is associated with the development of metastasis and worse outcome in P-NETs. Novel therapeutic approaches by targeting STAT3 or even IL-6 can be an alternative for these patients. Another interleukin associated with antiangiogenic treatment is IL-8, which is associated with a very poor outcome. The use of IL-8 neutralizing antibodies in P-NETs has been proposed, since it has been used successfully in ccRCC cell lines.

Lysosomal sequestration is another well-known mechanism of resistance to antiangiogenic agents, which can be overcome with the use of P-gp inhibitors and alkalinizing agents. Also, the mTOR pathway involvement opens the possibility to consider combining these treatments, though it has a high associated toxicity.

Overexpression of EZH2 and its resistance to associated antiangiogenic agents show a dynamic behaviour of the tumor, since it can be overcome with dose escalation. Also, using a specific inhibitor may be an option, since it acts as a synergic agent with sunitinib and avoids the development of such resistance.

PlGF is found to be elevated in P-NETs patients, being associated with a poor outcome. Furthermore, it is also a mechanism of resistance to antiangiogenics; these data make it an important target to investigate and to take into account as a possible prognostic and response marker.

Switching to another TKI when resistance to sunitinib occurs is a practical clinical strategy to consider, but is not feasible nowadays outside clinical trials since sunitinib remains the only targeted therapy approved, so there is an urgent need for promoting the development of new generation TKIs in this setting, mainly considering the promising results from preliminary phase II trials.

Summarizing, angiogenesis is a tremendously complex process that implies central cell pathways, not only related to VEGFR but also to other tyrosine kinases receptors or even signalling routes as EMT, cell survival, autophagy, or apoptosis. It is also important to consider that TME plays a central role in tumor regulation, hypoxia, and the relationship between tumor cells and immune system. That is why the angiogenic regulation with only one therapeutic agent is difficult and probably insufficient if we attend to all the cross-talking in this complicated system.

## 8. Conclusions

P-NETs are a rare group of malignancies with a distinctive behaviour. The better knowledge of its molecular biology over the last few years has made them an interesting field of research. Sunitinib is the only targeted therapy approved for this type of tumor, and its mechanisms of resistance need to be further clarified in order to be able to understand its behaviour in P-NET to optimize the treatment sequence for these patients. Furthermore, there are several ongoing clinical trials with other targeted therapies, as well as different therapeutic approaches, with promising results in preclinical models and clinical trials.

## Figures and Tables

**Table 1:** Classification differences in the last decade. HPF: High power field. WD NETs: well-differentiated neuroendocrine tumors.

WHO 2010	Mitotic Count	Ki67 Index	Previous
WD NETs G1 WD NETs G2	<2 × 10 HPF	≤2%	G1
	2–20 × 10 HPF	3–20%	G2
PD NEC G3	>20 × 10 HPF	>20%	G3
MANEC			
WHO 2017	Mitotic Count	Ki67 index	
WD NETs G1 WD NETs G2	<2 × 10 HPF	<3%	
	2–20 × 10 HPF	3–20%	
WD NETs G3	>20 × 10 HPF	>20%	Difference is made upon molecular and histological features
PD NEC G3	>20 × 10 HPF	>20%	
MINEN	To qualify as MENEN each component (endocrine and non-endocrine) must have at least 30%		

PD NEC: poorly-differentiated neuroendocrine cancer. G1: grade 1. G2: grade 2. G3: grade 3. MANEC: (Mixed adeno-neuroendocrine carcinoma). MINEN (Mixed endocrine non-endocrine neoplasms).

**Table 2:** Main characteristics and treatment options from patients included in the phase III trials evaluating the role of SSA in WD NETs.

Localization		Midgut		Pancreas		Liver Tumor Burden High (>25%)
Grade of Differentiation		G1	G2	G1	G2	
Ki 67		<2%	2–10%	<2%	2–10%	
First line SSA treatment	Octreotide LAR	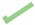				
	Lanreotide Autogel	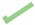	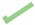	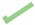	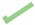	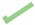

SSA: Somatostatin analogue, IFN: interferon.

**Table 3:** Clinical trials evaluating the activity of antiangiogenic agents in NETs. [18,19,20,21,22,23].

	Sunitinib		Cabozantinib		Lenvatinib		Pazopanib	
**Trial design**	Phase II_NR	Phase III_R	Phase II_NR	Phase II_NR	Phase II_NR	Phase II_NR	Phase II_NR	Phase II_R
**Primary tumor origin**	Carcionid 41	pNET 171	GI NET 41	pNET 20	GI NET 56	pNET 55	GEPNET	Carcinoid 97 + 74
**Follow-Up (m)**	15.1	60	23.3				17	44
**Previous treatment (SSA + others) (%)**	53.7 + 44	35 + 66	98 + NA 1 (0–6)	75% + NA 3 (0–8)	98 + 0	84 + 100	82 + 100	94 + 26
**ORR (%)**	2.4	9	15	15	16.3	42.3	9.5	2.1 vs. 0
**SD (%)**	82.9	63	75 (10% UK)	63 (17% UK)	74	50	50.0	72.2 vs. 73.0
**PD (%)**	2.4	14	0	5	0	0.02	40.5	4.1 vs. 18.9
**mPFS (m)**	10.2	11.4 vs. 5.5	31.4	21.8	15.4	15.53	9.5	11.6 vs. 8.5
**mOS (m)**	25.3	38.6 vs. 29.1	NA	NA	NA	NA	NA	41.3 vs. 42.4

NR: not reported. R: reported. NA: not available.
